# Biologically Plausible Class Discrimination Based Recurrent Neural Network Training for Motor Pattern Generation

**DOI:** 10.3389/fnins.2020.00772

**Published:** 2020-08-12

**Authors:** Parami Wijesinghe, Chamika Liyanagedera, Kaushik Roy

**Affiliations:** School of Electrical and Computer Engineering, Purdue University, West Lafayette, IN, United States

**Keywords:** echo state networks, separation property, approximation property, class discrimination, motor pattern generation

## Abstract

Biological brain stores massive amount of information. Inspired by features of the biological memory, we propose an algorithm to efficiently store different classes of spatio-temporal information in a Recurrent Neural Network (RNN). A given spatio-temporal input triggers a neuron firing pattern, known as an attractor, and it conveys information about the class to which the input belongs. These attractors are the basic elements of the memory in our RNN. Preparing a set of good attractors is the key to efficiently storing temporal information in an RNN. We achieve this by means of enhancing the “separation” and “approximation” properties associated with the attractors, during the RNN training. We furthermore elaborate how these attractors can trigger an action via the readout in the RNN, similar to the sensory motor action processing in the cerebellum cortex. We show how different voice commands by different speakers trigger hand drawn impressions of the spoken words, by means of our separation and approximation based learning. The method further recognizes the gender of the speaker. The method is evaluated on the TI-46 speech data corpus, and we have achieved **98.6%** classification accuracy on the TI-46 digit corpus.

## 1. Introduction

The biological brain continues to be one of the most astounding enigmas of nature. Unearthing the brain's mysteries for inspiration is prevalent in recent artificial neuron modeling efforts. For instance, spiking neural networks have gained attention over the years due to their information representation with biological neurons (Davies et al., 2018; Wijesinghe et al., 2018). Owing to spike based inter-neuron communication, the brain has evolved to achieve its signal-processing capabilities, at a power consumption which is orders of magnitude smaller than the state-of-the-art super computers (Cruz-Albrecht et al., 2012). Similar to the spike based communication, the “memory” is another important aspect that makes the biological brain fascinating. Memory is the information stored inside the brain by tuning synaptic weights through supervised and unsupervised learning transpired over a duration of time (Reber, 2010). A human brain can typically store information worth ~2.5 petabytes, which is equivalent to the amount of data telecast through a television over 300 years (Nabavi et al., 2014). Other recent studies have shown that it could potentially be even 10-folds higher than what it was estimated, due to the discovery of 26 distinguishable synaptic strengths (Bartol et al., 2015). In contrast to digital memories, the content inside the brain is not byte-addressable (Forsythe et al., 2014). Instead, the content operates within a dynamic dictionary that constantly shifts to make room for new meaning (Forsythe et al., 2014).

The memory of the biological brain is fundamentally associative. As hypothesized and based on experiments conducted on monkeys (Suzuki, 2007), the hippocampus is important for the early formation of the new associations in memory. A new piece of information can be absorbed well if it can be associated to an existing knowledge that is already anchored in the memory. For example, if one wants to learn a new word called *rubeus* in Latin, which means “red,” he/she can potentially think about the “r” sound at the beginning of both the words. Here the word “red” is in the existing memory and sound “r” is the association to the new word. The person can now easily remember that *rubeus* means red. Finding an association to an existing content is not merely sufficient to properly remember new data. For instance, consider the same previous word *rubeus*. The person who just remembered the association of the “r” sound will only be able to answer the question “which color is *rubeus* in Latin?,” but not “what is ‘red' in Latin?.” If one does not remember the actual word, the answer to question “which color is *ravus* in Latin?” would again be “red” since the person merely remembers some association with the sound “r.” The answer is incorrect since *ravus* means gray. In a more complicated situation, assume one should remember the word *rot* which is red in German along with *rubeus*. Now the person should consider ways of distinguishing the two words despite the fact that they have the same meaning “red,” in order to properly digest them simultaneously.

In this work, we consider the above phenomenon related to memory and construct an algorithm to help store significant amounts of data in a neural network. The brain is capable of remembering both static (example an image) and temporal (example a song) information. We will be focusing on the latter form of data learning for a recurrent neural network. One hypothesis for the way the brain stores temporal information is by means of attractors (Laje and Buonomano, 2013). This hypothesis is built upon the functionality of the cerebellum: a part of the biological brain that plays an important role in maintaining correct timing of motor actions. The role of cerebellum in sensory-motor actions is explained by means of experiments conducted on cerebellar patients (Jacobson et al., 2008). Such patients have increased temporal variability between motor actions, such as inaccurate timing of ball release when throwing a ball (Timmann et al., 2001) or variability shown during rhythmic tapping (Ivry et al., 1988). Cerebellum is also known for using associative learning to pair external stimuli with motor timing tasks (Paton and Buonomano, 2018). The classical eyeblink conditioning experiment shows how associative learning is used to program the cerebellum to react to a conditional stimulus such as a tone with an eyeblink reflex (Medina and Mauk, 2000; Johansson et al., 2016). This experiment is a perfect demonstration of the cerebellum's capacity for temporally specific learning. There are many standing theories as to how the cerebellum generates these temporal patterns and one such theory is the aforementioned attractor hypothesis (Laje and Buonomano, 2013). In this work we implement a biologically plausible reservoir computing (Wang and Li, 2016; Tanaka et al., 2019) network that uses this attractor hypothesis to emulate the temporal pattern generation capabilities of the cerebellum.

The temporal inputs that belong to a particular class trigger a certain internal neuron firing pattern. These patterns can be thought of as a representation of the existing knowledge in the memory corresponding to the temporal input. Let us call these anchored knowledge (or the internal dynamics of the network) as class attractors. The validity of the “attractor” hypothesis for large amounts of data and classes is yet to be analyzed. For instance, the work in Laje and Buonomano (2013) shows motor pattern generation application for voice commands but the number of inputs and classes are limited. As the number of different pattern classes increases, the corresponding class attractors are more likely to stay close to each other leading to more misclassifications. For example, one might mishear the word “bold” as “bald.” Here we propose a mechanism to enhance the deviation between the attractor dynamics by extracting key differences between input pattern classes.

In order to recognize whether a projection of a particular input is a better representation (in the context of a classification task), certain properties must be considered. Two such properties are “separation” and “approximation.” These are analogous to the phenomenon described previously on associativity in the biological memory. In a classification problem, projections of inputs corresponding to two different classes must stay apart from each other (separation). The projections that belong to the same class must stay close to each other (approximation). For instance, an utterance of the word “one” by male speakers should converge to one attractor (approximation). When this particular attractor is triggered, brain recognizes it as the word “one.” If the same word spoken by females also triggers the same attractor, then the brain will not be able to recognize whether the speaker is female or male, despite the fact that it could recognize the spoken word. Therefore, in a scenario where the gender of the speaker must be identified, the attractor triggered by the male speakers and female speakers for the same word should be different (separation). Closer the attractors are, harder it would be to recognize the gender of the speaker. Our proposed learning approach (for a recurrent neural network) takes into account these properties, and improves class discrimination for better accuracy in a sensory motor task. i.e., we convert utterances of words (sensory data) into handwritten impressions (motor action) using reservoir computing. The network furthermore recognizes the gender of the speaker, and generates an impression of letter “f” (for female) or letter “m” (for male).

In the context of temporal information processing, one can find numerous studies investigating the speech recognition problem using strictly feed forward networks such as Convolutional Neural Networks (Swietojanski et al., 2014; Palaz et al., 2015), Deep Neural Networks (Hinton et al., 2012), Hidden Markov Models (Tran and Wagner, 1999), and Spiking Neural Networks (Liu et al., 2019; Zhang and Li, 2019). Recently, biologically inspired training methodologies (Neftci et al., 2016) and reservoir computing solutions such as Liquid State Machines (Wang et al., 2015; Jin and Li, 2017) or Echo State Networks (ESN) (Skowronski and Harris, 2007; Laje and Buonomano, 2013) are been investigated extensively as an effort to bridge the gap between biological plausibility and practicality. Similarly, the work presented here are more geared toward replicating the activity of the cerebellum in generating complex motor patterns. We employ an ESN configured as a bio plausible practical implementation on how the cerebellum performs complex motor timing tasks. Like the cerebellum, the proposed network reacts to a temporal input and generates a timed motor response using a single reservoir network. A strictly feed forward network would be sufficient for the task if the objective was to simply classify the audio inputs into classes. However, in order to generate pre-determined timed responses such as motor tasks, a feed forward network would require additional timers and memory elements to store the sequences of movements to be performed.

An ESN is a simple form of a recurrent neural network with a pool of randomly interlinked leaky integrate analog neurons called the reservoir (Jaeger, 2007). The time varying inputs are connected to the reservoir by means of synapses of random weights. The reservoir neuron dynamics are directed toward a set of output neurons by means of a readout. These readout connections are trained using supervised methods. Some architectures use feedback connections from the output neurons to the reservoir neurons. However, in this work we do not use such feedback connections. In addition to training the readout connections, we also tune the input-reservoir connections and the recurrent connections within the reservoir itself.

The training mechanism consists of three major steps. 1. Separation based input-reservoir connection training, 2. Approximation based innate dynamic training of the reservoir connections, and 3. Readout connection training for motor pattern generation. During the first step, we obtain a set of well-separated innate dynamics per class (class attractors). Then in the second step, we converge all the reservoir dynamics of inputs in a given class, to its corresponding class attractor. Finally we convert the reservoir dynamics to a set of time varying coordinates to generate an impression of the spoken word, by means of the readout layer. We employ the entire TI46-digit and alphabet corpuses for our experiments. Following are the key contributions of this work.

1. Explaining the need of a set of well-separated attractors when dealing with bigger data sets.
2. Proposing a training algorithm to initially separate the attractors, and then make the reservoir dynamics for input instances, converge to their corresponding class attractor (discrimination based training).
3. Using two full data sets, validate how the accuracy improved with the separation based training.
4. Show the ability to generate motor patterns based on other attributes of the inputs. Apart from drawing the spoken character, the trained ESN can now recognize the gender of the speaker and generate a motor pattern corresponding to that simultaneously.
5. Use the network on an image based application to show the generality of the discrimination based training method.

## 2. Materials and Methods

### 2.1. Echo State Networks—The Network Structure

**Figure 1:**
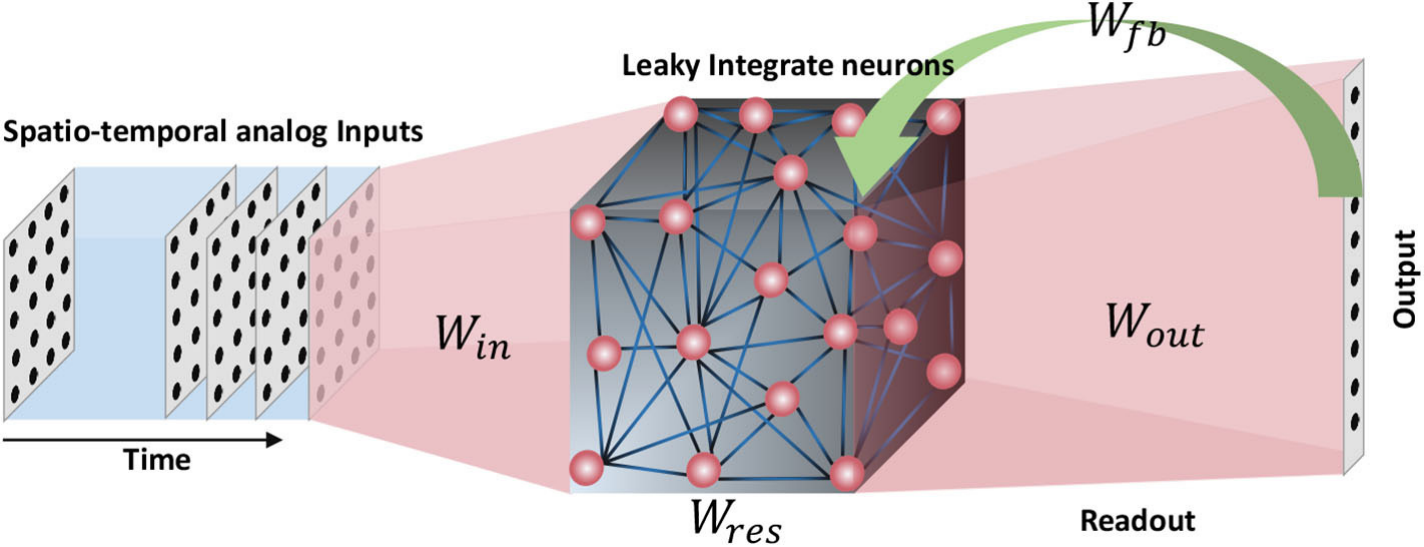
The structure of an echo state network. A pool of randomly interlinked neurons known as the reservoir is the major component of an ESN. The neurons are analog leaky integrate neurons. Time varying inputs are connected to the reservoir and the reservoir neuron dynamics are decoded at the end of the readout. Some ESN architectures have feedback connections from the output to the input.

In this section, the structure of the recurrent neural network involved in this work will be explained. For spatio-temporal data processing, we used an echo state network, a simple form of a recurrent neural network architecture (when compared with Long Short Term Memory networks or LSTMs; Hochreiter and Schmidhuber, 1997). An ESN (Jaeger, 2007) consists of a pool of neurons recurrently interlinked, called the reservoir, and a readout layer. Inputs are applied on the reservoir neurons by means of input-to-reservoir connections. Owing to the recurrent connections within the reservoir, a temporally varying input signal applied on the network at time *t* = 0, could potentially leave the neurons firing (an “echo” of the input) even after the input has been detached (hence the name echo state network). Such “echoes” or residuals of the inputs can be measured through the output layer in order to perform a particular task. The output connections are typically trained using supervised methods such as delta rule, backpropagation (Rumelhart et al., 1988) and recursive least square algorithm (RLS) (Haykin, 1991). Some architectures (Tanaka et al., 2019) also have a set of feedback connections from the output to the reservoir (Figure 1). There have been multiple opinions on whether the brain acts as a feedback system, and according to studies (Byrne and Dafny, 1997), the brain is mostly a feedforward system. Feedforward systems are fast and require certain knowledge about the outcome that correspond to a given input (similar to a lookup table). On the other hand, systems with feedbacks continuously monitor the output in order to modify the internal dynamics to achieve a certain target output. Such systems are sluggish than feedforward systems. Therefore, with the goal to achieve faster training, we did not use the feedback connections in our structure.

#### 2.1.1. Reservoir Neurons

The neurons within the reservoir are leaky integrate neurons (Jaeger, 2007). The dynamics of the neurons are analog in fashion and can be given by the following equation.

(1) $$\begin{array}{r} -\tau_{uni}\frac{dx\left(t\right)}{dt}=-\alpha_{l}x\left(t\right)+W_{res}r\left(t\right)+W_{in}u\left(t\right)\\ \;r\left(t\right)=tanh(x\left(t\right)) \end{array}$$

where *x*(*t*) is the state of the neuron, *r*(*t*) is the firing rate of the neuron (output of the neurons, which is simply a non-linear function of the neuron's state), $W_{res}\in {\mathbb{R}}^{n_{res}\times n_{res}}$ is the connection matrix inside the reservoir, and $W_{in}\in {\mathbb{R}}^{n_{i}\times n_{res}}$ is the connection matrix from the inputs [*u*(*t*)] to the reservoir. τ_*uni*_ is the uniform time constant, and α_*l*_ is the leak coefficient. The output of the network is taken from the readout as follows.

(2) $$\begin{array}{r} y\left(t\right)=W_{out}r\left(t\right) \end{array}$$

where $W_{out}\in {\mathbb{R}}^{n_{res}\times n_{out}}$ is the connection matrix from reservoir neurons to the outputs. The constant parameter values were selected as proposed in literature (Laje and Buonomano, 2013) and certain parameter were swept till the highest accuracy was achieved for a given number of neurons. The values are illustrated in Table 1. For solving the differential equations, we used standard Heun's method (Süli and Mayers, 2003) with a time step (*dt*) of 1 ms.

**Table 1:** The hyper-parameters and their values used in this work.

**Parameter name**	**Value**
Sensory phase	300 ms
Motor phase	300 ms
Uniform time constant	0.04 ms
Leak constant	0.8
Time step	1 ms
Input channels	39
Output dimensions	2
Spectral radius scaling factor	1.4
Inverse learning rate (separation)	500
Inverse learning rate (approximation)	100

#### 2.1.2. Network Connections

In a conventional echo state network, the reservoir and input-to-reservoir connections are randomly generated and only the final readout weights are trained. However, all the connections in the network in this work are trained using RLS learning rule. In a reservoir with randomly initialized weights (i.e., when no learning is involved to tune the connections), it is a good practice to have sparsity within the network in order to get better projections of the inputs. For example, multiple sparsely connected small reservoirs can give better class discrimination (hence better accuracy) for spatio-temporal data classification tasks using reservoir computing (Wijesinghe et al., 2019). This is due to the fact that different combinations from the same set of inputs were fed to the readout by means of an ensemble of reservoirs. However, in this work, since we are training all the network connections, we left the percentage connectivity equal to 100%. This gives more number of hyper parameters to change and finding the optimum set of weights is much faster using the RLS method (Sussillo and Abbott, 2009). Before training, the input-reservoir connections and within reservoir recurrent connections were randomly initialized using a normal Gaussian distribution. The reservoir connections were scaled by a factor in such a way that the spectral radius of the connection matrix is *r*_*s*_ = 1.5 (Laje and Buonomano, 2013).

### 2.2. Application

We perform a sensory motor application where the sensory input data are utterances of words, and the outputs are hand drawn impressions related to the spoken word and the speaker. For example, if the input voice command is an utterance of “six” by a female speaker, the output motor action would be to draw digit 6, and a letter “f.” The inputs words are either utterances of digits or letters in the alphabet. In order to show the generality of our training method, we further included a third application that does not involve voice as an input command. In this application, the input is a hand drawn image, and the output is a time sequence that can be used to draw the corresponding digit. It further generates a letter “i” or “n” as another output at the same time, depending upon the face of the drawn digit (“*i*” for italic, “*n*” for normal character face). Refer to the Supplementary Materials for further details and results on this application.

#### 2.2.1. Inputs

The first step is converting the input commands to a proper format to be processed by the network. For the input voice commands, the audio samples available in wave format were preprocessed based on Lyon's Passive Ear model (Lyon, 1982) of the human cochlea, using Slaney's MATLAB auditory toolbox (Slaney, 1998). The model was used to convert each audio sample to temporal variation in the amplitude of 39 frequency channels. The 39 signals were then down sampled (× 4) in the temporal axis and applied as the input to the reservoir. The time during which the input data is applied on the network is the “sensory phase.”

The two temporal (speech) data sets used in this work are:

1. Digit sub-vocabulary of the TI46 speech corpus (Liberman et al., 1993) (TI-10)
2. TI 26-word “alphabet set”; a sub-vocabulary in the TI46 speech corpus (Liberman et al., 1993) (TI-alpha).

TI-10 consists of utterances of the words “zero” through “nine” (10 classes) by 16 speakers. There are 1, 594 instances in the training data set and 2, 542 instances in the testing data set. TI-alpha, on the other hand, has utterances of the words “A” through “Z” (26 classes). There are 4, 142 and 6, 628 instances in the training and testing data sets, respectively.

#### 2.2.2. Outputs

**Figure 2:**
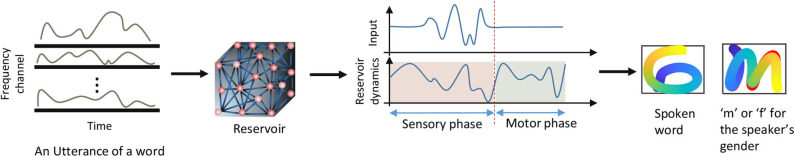
The inputs and outputs of the recurrent network. The network dynamics are divided in to two phases. Sensory phase during which the input is applied, and the motor phase during which two motor patterns are generated.

At the end of the sensory phase, the residual dynamics in the reservoir are converted to time varying signals at the output by means of a readout layer. The readout layer gives two sets of time varying *x* and *y* coordinates of hand drawn impressions (Figure 2). For the applications where the input is a set of voice commands, one such *x* and *y* coordinate set recognizes the gender of the speaker and draw either “f” (if female) or “m” (if male) accordingly. The other coordinates set generates the hand drawn impression of the uttered digit or letter. The duration within which these impressions are drawn is the “motor phase.” The motor phase begins just after the sensory phase.

The network presented in this work is different from other networks that are used for traditional identification problems. The output of our network does not specifically say what class the input belongs to. The network responds to a spatio-temporal input with a spatio-temporal output based on prior knowledge, and the observer performs the classification task when they are reading the output. If an input that does not belong to any of the trained classes is presented to the network, the network can produce some temporal pattern that is not recognizable by any observer. Hence this is an open-set problem because the output can take infinitely different forms.

## 3. Results

### 3.1. Training Methodology

**Figure 3:**
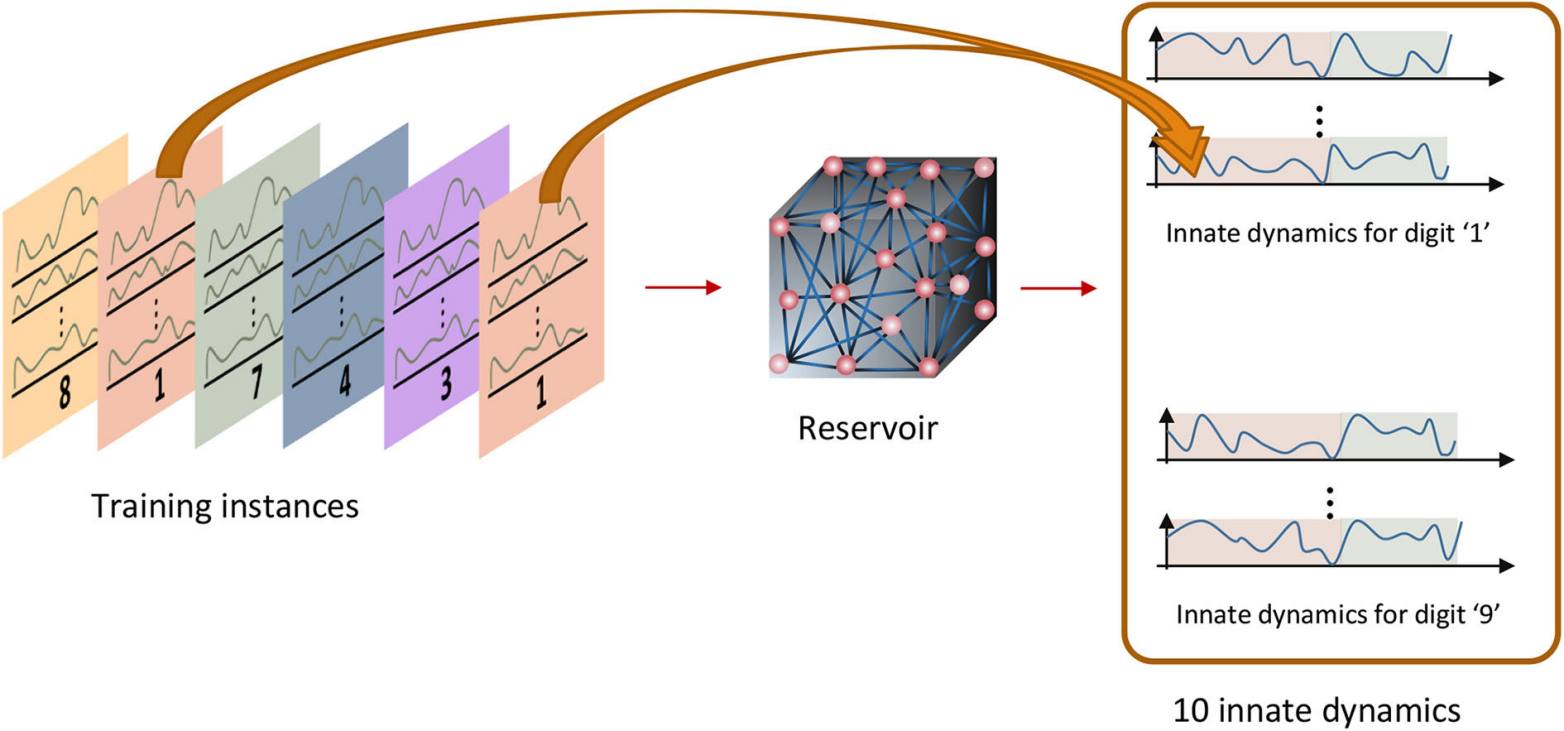
Innate training: the instances that belong to one class should converge to a pre-assigned attractor.

The temporal inputs applied during the sensory phase trigger the neurons to fire in a certain way during the motor phase. The goal is to activate the same neuron firing pattern when inputs in a particular class are fed. i.e., there must be a specific firing pattern per class as shown in Figure 3. These reservoir neuron firing patterns are called the “class attractors.” The key idea of the training methodology is to create a good set of class attractors by means of changing the input-reservoir and reservoir-reservoir connections, and changing the reservoir-readout connections to draw the corresponding impression. Following subsections will explain how the weights are systematically changed to craft these attractors. The entire training process has three major steps as explained below.

#### 3.1.1. Step 1 : Separation Based Input-Reservoir Connection Training

The first step is creating a set of proper attractors which are triggered by input instances that belong to different classes. In order to assign an initial value to the class attractors, a set of inputs that represent each class (or class-template inputs) in the data set is required. We categorize the instances in the data set by both the spoken word and the gender of the speaker. For example, the TI-10 data set contains utterances of words by 8 male speakers and 8 female speakers, and each speaker utters the same word multiple times. Here the word “six” spoken by female speakers is considered as one class (notified as *Class*_6, *f*_), and the word “six” spoken by male speakers is considered as another class (notified as *Class*_6, *m*_). This class assignment is done since the readout layer recognizes both the spoken word and the gender of the speaker. Therefore, the total number of classes assigned for the TI-10 dataset is 20 (10 digits × 2 genders). Similarly, the total number of classes assigned for the TI-alpha dataset is 52 (26 alphabet letters × 2 genders).

A set of class-template inputs are created by taking the mean value of all the instances in each class. For example, assume there are *f* frequency channels, *n*_*T*_ number of time steps in the sensory phase, and *n*_*f*_ number of female speakers speaking the word “six” *i* times each. This gives *n*_*f*_ × *i* number of 2 dimensional (*f* × *n*_*T*_) examples in *Class*_6, *f*_. The average 2 dimensional input among these *n*_*f*_ × *i* examples is evaluated and assigned as the input template of the particular class.

The generated class-template inputs are then applied on the reservoir to obtain the “innate dynamics” (“innate dynamics” are the firing rate dynamics of the neurons in the reservoir, for an applied input, under zero initial conditions and in the absence of noise) that can be considered as the initial assignment for class attractors. The work in Goudar and Buonomano (2018) uses these innate dynamics as the final class attractors (without any modifications), and the reason behind using the dynamics inherently generated by the reservoir as the attractors is not evident.

The difference among the innate dynamics indicate how separated the class attractors are. If two class attractors are close to each other, it is more likely that some input instances that belong to one class can trigger an attractor that corresponds to the other class, instead of converging to the correct class attractor. This will lead to erroneous classification, or improper motor pattern generation at the readout layer. When the number of classes and examples are higher, the possibility of two attractors staying close to each other in the multi-dimensional space is higher. Hence there is a need for separating the class attractors.

In order to separate the class attractors, a quantitative measure of separation is required. Multiple measures for separation (a measure of “kernel quality”) in reservoirs are available in literature. Two such key ways of quantifying separation are known as *pairwiseseparationproperty* and *linearseparationproperty* (Maass et al., 2005; Legenstein and Maass, 2007; Wang et al., 2015). The pairwise separation property is defined as the distance between two continuous time states of a reservoir [*x*_*u*_(*t*) and *x*_*v*_(*t*)], resultant from two separate inputs *u*(*t*) and *v*(*t*). The distance can be calculated by the Euclidean norm between *x*_*u*_(*t*_*n*_) and *x*_*v*_(*t*_*n*_) at sample point *t*_*n*_. The average across all the sampled instances (∀*t*_*n*_) can be used to evaluate the final pairwise separation property, as explained in the following equation

(3) $$\begin{array}{r} SP_{PW}=\frac{1}{N_{samples}}\overset{N_{samples}}{\underset{\text{n }=\;1\left(0<t_{n}<T\right)}{\sum }}||x_{u}\left(t_{n}\right)-x_{v}\left(t_{n}\right)|| \end{array}$$

where *N*_*samples*_ is the number of sample points. The pairwise separation property (*SP*_*PW*_) can be used as a measure of the separation property for two given inputs. However, most real-life applications deal with more than two input spike trains. To address this, linear separation property is proposed as a more suitable quantitative measure to evaluate the reservoir computational power (Maass et al., 2005; Legenstein and Maass, 2007; Wang et al., 2015). The linear separation property (*SP*_*lin*_) is the rank of the *N* × *m* matrix *M*_*S*_, which contains the continuous time states [*x*_*u*_1__(*t*_0_), ..., *x*_*u*_*m*__(*t*_0_)] of the reservoir as its columns. The continuous time state *x*_*u*_*i*__(*t*_0_) is the reservoir response to the input *u*_*i*_ (from the training set), at time *t* = *t*_0_. If the rank of the matrix is *m*, it guarantees that any given assignment of target outputs $y_{i}\in {\mathbb{R}}^{N_{out}}$ at time *t*_0_ can be attained by means of a linear readout (Maass et al., 2005). The rank of *M*_*S*_ is the degree of freedom the linear readout has, when mapping *x*_*u*_*i*__ to *y*_*i*_. Even though the rank is <*m*, it can still be used as a measure of reservoir quality(Maass et al., 2005).

(4) $$\begin{array}{r} M_{S}=\left[x_{u_{1}}\left(t_{o}\right),...,x_{u_{i}}\left(t_{o}\right),...,x_{u_{m}}\left(t_{o}\right)\right]\\ SP_{lin}=rank\left(M_{S}\right) \end{array}$$

However, it is noteworthy that when the number of reservoir neurons is much larger than the number of inputs that is required to be separated (*N* ≫ *m*), the rank of the matrix *M*_*S*_ is most likely equal to *m* (*SP*_*lin*_ = *m*). Furthermore, *SP*_*lin*_ is a discrete function and two reservoirs having the same *SP*_*lin*_ does not necessarily mean that their separation capability is identical. It is also noteworthy that the reservoir responses to *m* inputs can be further separated, even though the *SP*_*lin*_ has reached its highest possible value *m*.

In our work, it is required to increase the separation between the attractors. The number of attractors is equal to the number of classes, which is larger than two (*S*_*PW*_ is not applicable) and much smaller than the reservoir neurons (*SP*_*lin*_ is not applicable). The need for a quantitative measure of separation, that is a continuous function of the ESN weights arise. Therefore, we use insights from linear discriminant analysis (LDA) (Fisher, 1936; Fukunaga and Mantock, 1983; Hourdakis and Trahanias, 2013) to quantify the separation between the class attractors. The between class scatter matrix in the following equation contains information on how far each data point is located from the global mean, in the high dimensional space (Fukunaga and Mantock, 1983; Wijesinghe et al., 2019). Each data point is a vector that contains all the elements in an attractor matrix.

(5) $$\begin{array}{r} S_{b}=\overset{L}{\underset{i\;=\;1}{\sum }}P\left(\omega_{i}\right)\left(\mu_{i}-\mu_{g}\right){\left(\mu_{i}-\mu_{g}\right)}^{T} \end{array}$$

In the equation, μ_*i*_ is the sample mean vector (centroid) of class ω_*i*_, *P*(ω_*i*_) is the probability of class ω_*i*_, *L* is the number of classes, and μ_*g*_ is the global sample mean vector. The single measure that quantifies the separation is given by the trace of the above matrix (Wijesinghe et al., 2019).

(6) $$\begin{array}{r} SP=trace\left(S_{b}\right) \end{array}$$

Higher SP suggests better separation among the attractors. In the first step of the training process, we change the input-reservoir connections, such that the SP is increased. We use a modified version of the inverse of RLS for this purpose. The standard RLS learning rule, implemented according to the first-order reduced and controlled error (FORCE) algorithm can be used as follows to obtain a target dynamic in the reservoir (Sussillo and Abbott, 2009)

(7) $$\begin{array}{r} w\left(t\right)=w\left(t-\Delta t\right)-P\left(t\right)x_{in}\left(t\right)e\left(t\right) \end{array}$$

$w\left(t-\Delta t\right)\in {\mathbb{R}}^{n_{i}\times n_{res}}$ is the input-reservoir connection matrix before the weight update, $x_{in}\left(t\right)\in {\mathbb{R}}^{n_{i}}$ is the input dynamics at time *t*. Each weight update is done in Δ*t* time steps and it can be larger or equal to the simulation time step. The rule is similar to the delta rule but with multiple learning rates given by the matrix $P(=(x_{in}\left(t\right)x_{in}^{T}\left(t\right)+\alpha I{)}^{-1},\alpha$ is a constant), which is a running estimate of the inverse of the correlation matrix of *x*_*in*_(*t*) (Sussillo and Abbott, 2009). $e\left(t\right)\in {\mathbb{R}}^{n_{y}}$ is the error between the target *f*(*t*) and the actual reservoir dynamics at time *t*. *n*_*i*_, *n*_*res*_, and *n*_*y*_ are the number of input frequency channels, number of reservoir neurons and number of readout neurons, respectively.

(8) $$\begin{array}{r} e\left(t\right)=w^{T}\left(t-\Delta t\right)x_{in}\left(t\right)-f\left(t\right) \end{array}$$

**Figure 4:**
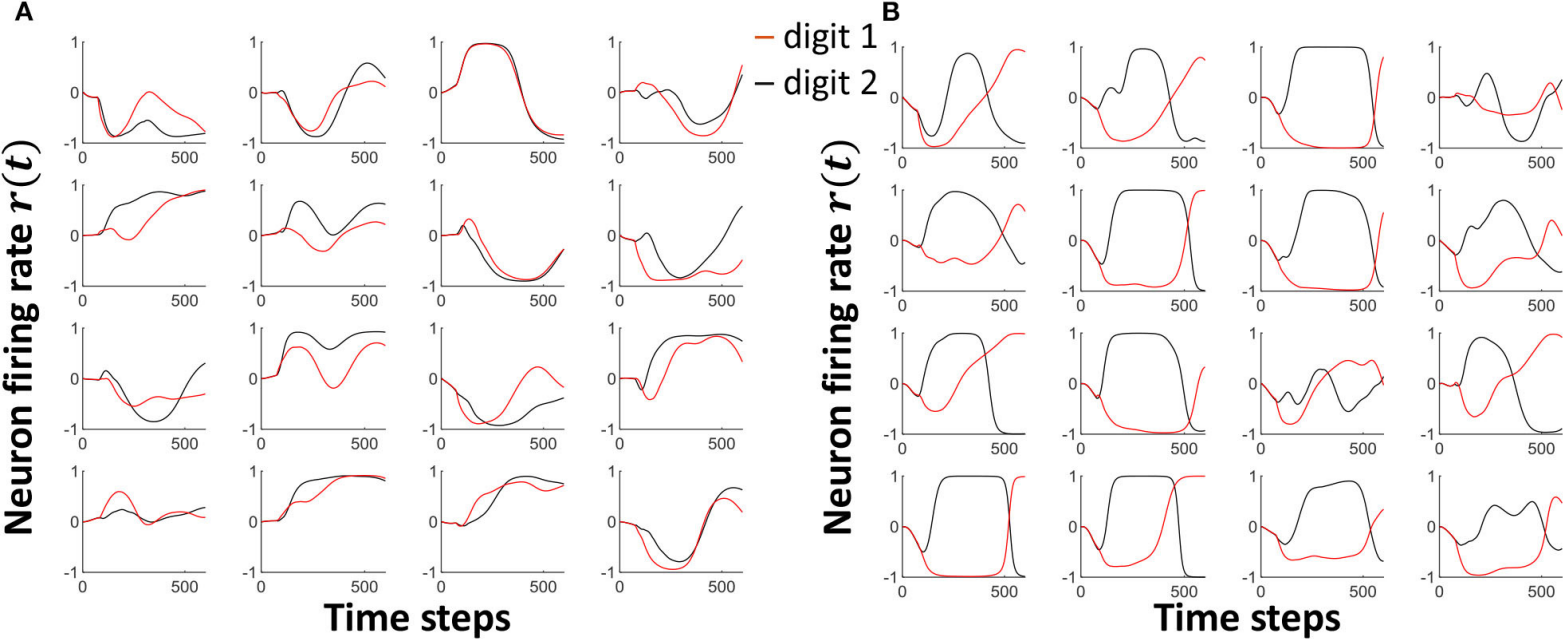
The neuron attractor dynamics of 16 randomly picked neurons for two input voice utterance classes, viz. *Class*_1, *f*_ and *Class*_2, *f*_. **(A)** The dynamics before separation enhancement training **(B)** The dynamics after separation enhancement training. Note that the separation between the class attractors have significantly improved.

The learning rule makes the dynamics of the reservoir to reach a target function *f*(*t*). However, the goal is to increase the distance between a set of attractors and to that effect, we modify the learning rule as follows. First we pick an input template of a particular class *i*, and apply it on the reservoir. The resultant reservoir dynamics of class *i* are then compared with previously evaluated attractors of class *j* (*j* = 1, 2, ...., *L*; *j* ≠ *i*) to evaluate the difference [*e*_*i, j*_(*t*)] between them. Ideally we expect this difference to be large to obtain a set of well separated attractors. Considering this, the weight update rule can be modified as follows.

(9) $$\begin{array}{r} w\left(t\right)=w\left(t-\Delta t\right)+\gamma P\left(t\right)x_{in}\left(t\right)(\vec{1}⊘e_{i,j}\left(t\right)) \end{array}$$

where $\left(\vec{1}⊘e_{i,j}\left(t\right)\right)$ gives the element-wise inverse of the error vector, and γ is a scaling factor. The input-reservoir weight update method extracts subtle differences in the input templates and exaggerates them, so that the differences are well-portrayed in the attractors. Figures 4A,B show how the attractors of *Class*_1, *f*_ and *Class*_2, *f*_ in the TI-10 dataset vary with time, before and after the weight update, respectively. Note that the separation between the two classes has visibly improved.

The main goal of the above elaborated first step (where we train the input-reservoir connections), is getting a set of well separated attractors to converge to. The attractors are directly dependent upon the applied inputs. If the input-reservoir weights were left randomly initialized and untrained, the contribution from the inputs will be random. Inputs from different classes can have different features. For instance, a female utterance of “*one*” can have more high frequency components than a male utterance of “*one*.” We need to enhance the contribution from these distinguishing features in the input, to the reservoir. If this step is done collectively with the reservoir weights, we will have more hyper parameters to optimize. Therefore, during collective weight training, the changes in input-reservoir weights will be miniscule (in order to achieve the same separation between attractors given by only training the input-reservoir weights). For instance, when only input weights were used to train during the first step, 51% of the weight changes (Δ*w*) were >±0.01. In contrast, when the input-reservoir and reservoir-reservoir connections were trained collectively, only 0.25% of the input weight changes were >±0.01. By training the input-reservoir weights separately, we get the best contribution from the input toward the reservoir and the optimum usage of input-reservoir weights which otherwise will be left almost untrained. Furthermore, collectively training the input -reservoir and reservoir-reservoir connections is computationally demanding. For instance, obtaining the attractors with collective weight training took 43 × more time, than the input-reservoir weight training, for approximately the same separation amounts.

#### 3.1.2. Step 2 : Approximation Based Reservoir Connection Training

**Figure 5:**
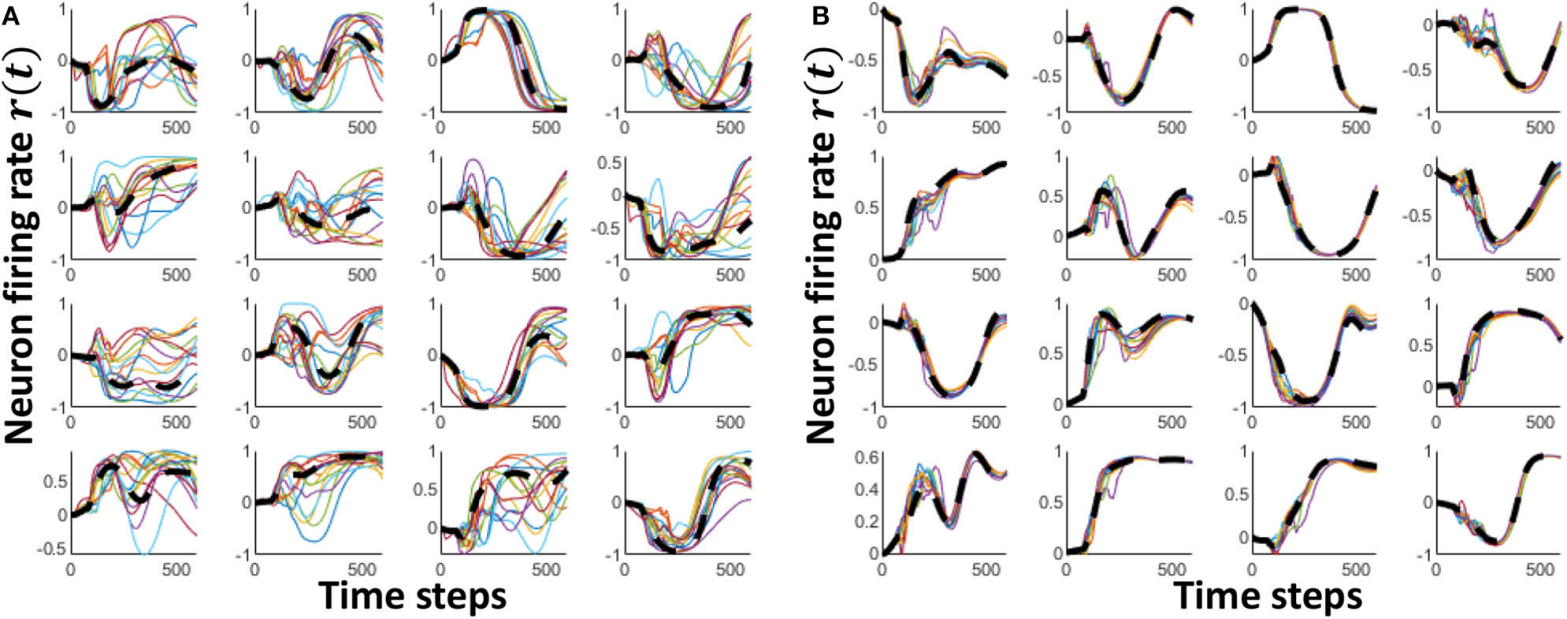
The neuron firing rate dynamics of 16 randomly picked neurons for multiple input voice utterances of “one.” **(A)** The dynamics before innate training **(B)** The dynamics after innate training. The black dashed line shows the innate dynamics of the same neurons for the class “one”.

After generating a well separated set of class attractors, the next step is converging all the instances in each class to their corresponding attractor. Figure 5A shows how the reservoir dynamics change for different instances in the same class. It is evident from the figure that all the instances in a class do not necessarily converge to the class attractor (shown in black dashed lines). To make them close to each other, here we train the reservoir-reservoir connections by means of the RLS rule implemented according to the FORCE algorithm (Sussillo and Abbott, 2009). The synaptic weight update is carried out as shown below.

(10) $$\begin{array}{r} w_{res}\left(t\right)=w_{res}\left(t-\Delta t\right)-P\left(t\right)r\left(t\right)e\left(t\right), \end{array}$$

where $w_{res}\in {\mathbb{R}}^{n_{r}es\times n_{r}es}$ is the connection matrix within the reservoir, *r*(*t*) gives the reservoir neuron firing rate at time *t*, *e*(*t*) gives the error between the actual reservoir dynamics and the corresponding attractor dynamic. *P*(*t*) is an *N* × *N* matrix updated along with the weights as follows

(11) $$\begin{array}{r} P\left(t\right)=P\left(t-\Delta t\right)-\frac{P\left(t-\Delta t\right)r\left(t\right)r^{T}\left(t\right)P\left(t-\Delta t\right)}{1+r^{T}\left(t\right)P\left(t-\Delta t\right)r\left(t\right)} \end{array}$$

The initial value of *P*(*t*) is selected as *P*(0) = *I*/α, where 1/α is the learning rate. As shown in Figure 5B, the reservoir dynamics are close to the class attractor after the training step. In this second step, we have achieved proper approximation.

#### 3.1.3. Step 3 : Readout Training for Motor Pattern Generation

In the previous two steps, we obtained a set of well separated attractors, and made all the instances of each class to converge to their corresponding class attractor. From these class attractors, now the readout layer generates two motor patterns that depict the gender of the speaker and the spoken word itself. Closer the reservoir dynamics are to their class attractors, easier it is for the readout layer to clearly generate the motor pattern.

**Figure 6:**
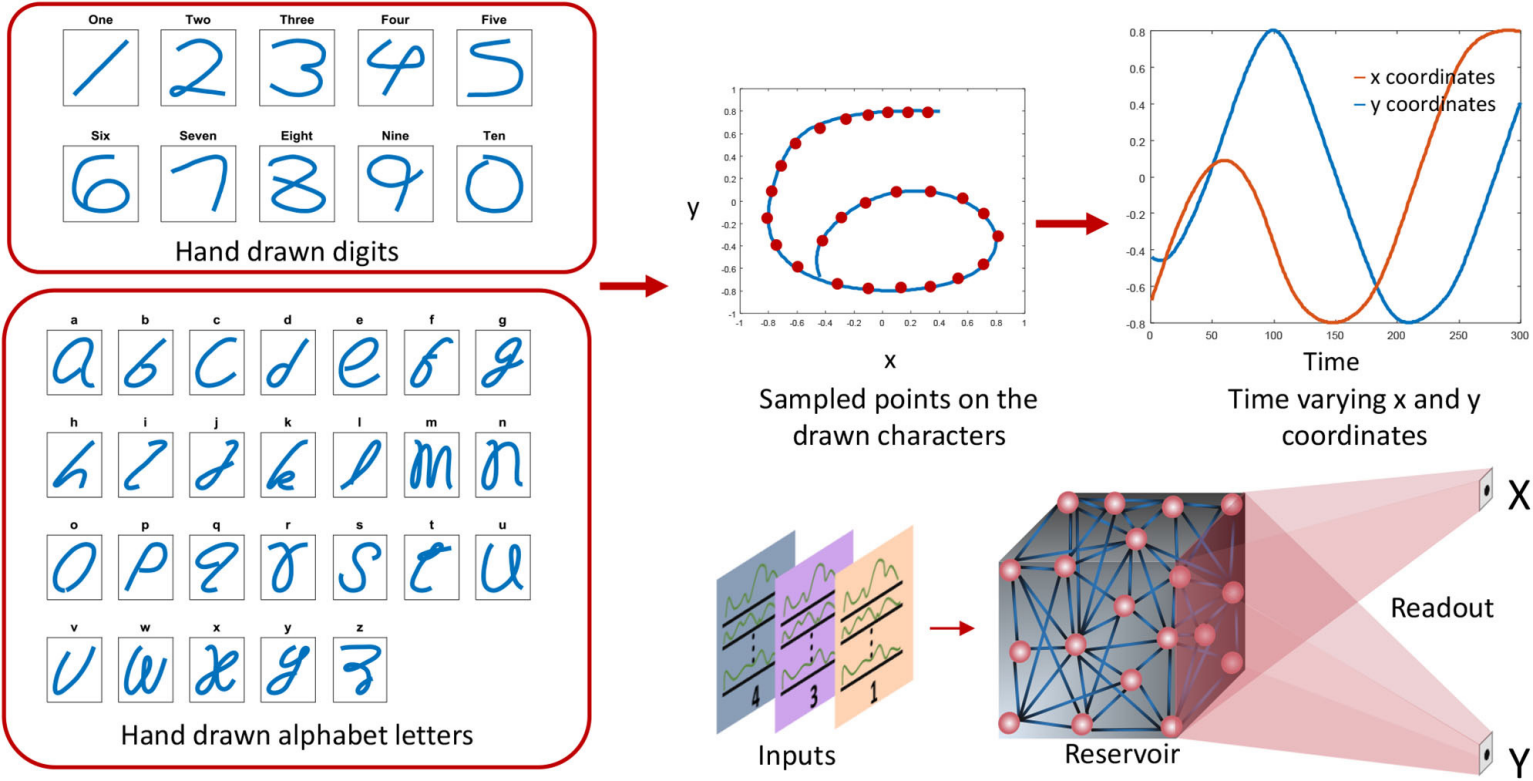
The preprocessing steps for the outputs. The hand drawn digits and alphabet letters are converted to x, y coordinates and arranged sequentially. Two x and y coordinate signals are generated to draw the corresponding character.

**Figure 7:**
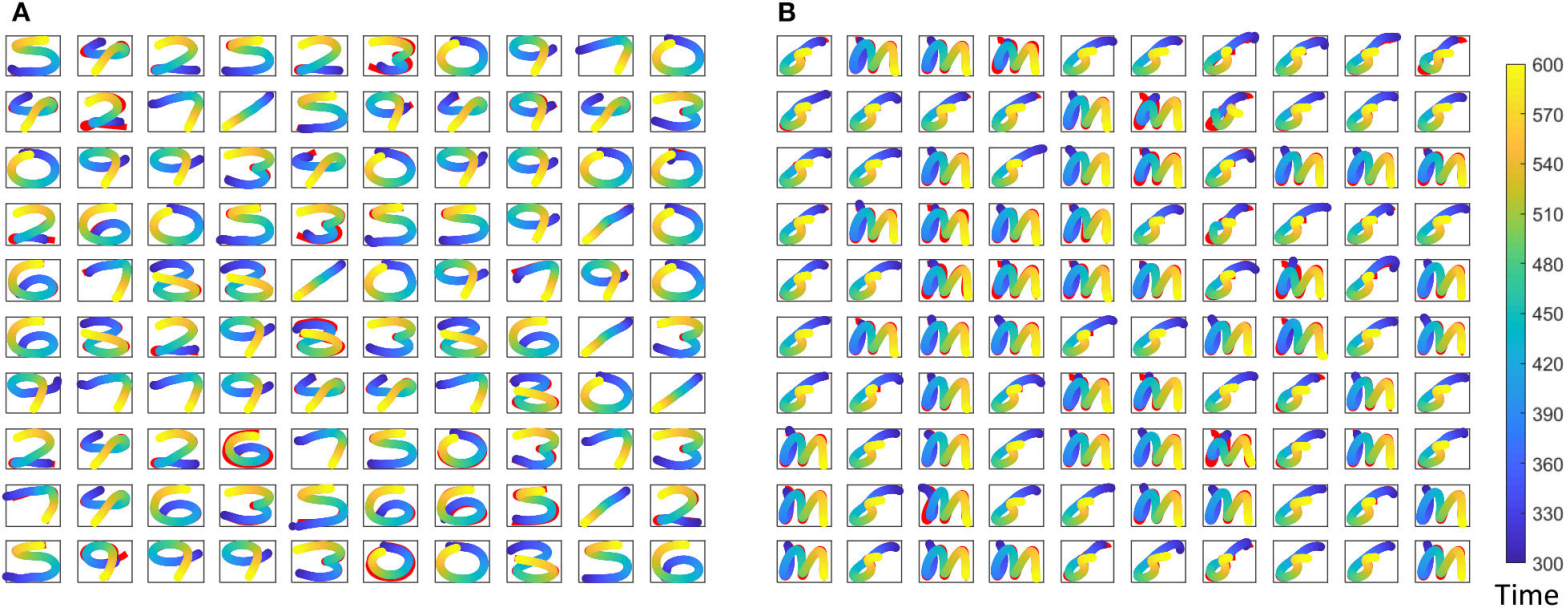
Different motor patterns generated by the ESN for randomly picked 100 input utterances from the TI-10 test data set. **(A)** The spoken digit in the input instance. **(B)** The gender of the speaker with “f” for female and “m” for male. Color code shows the time evolution of the signal and shown in red is the expected motor pattern.

**Figure 8:**
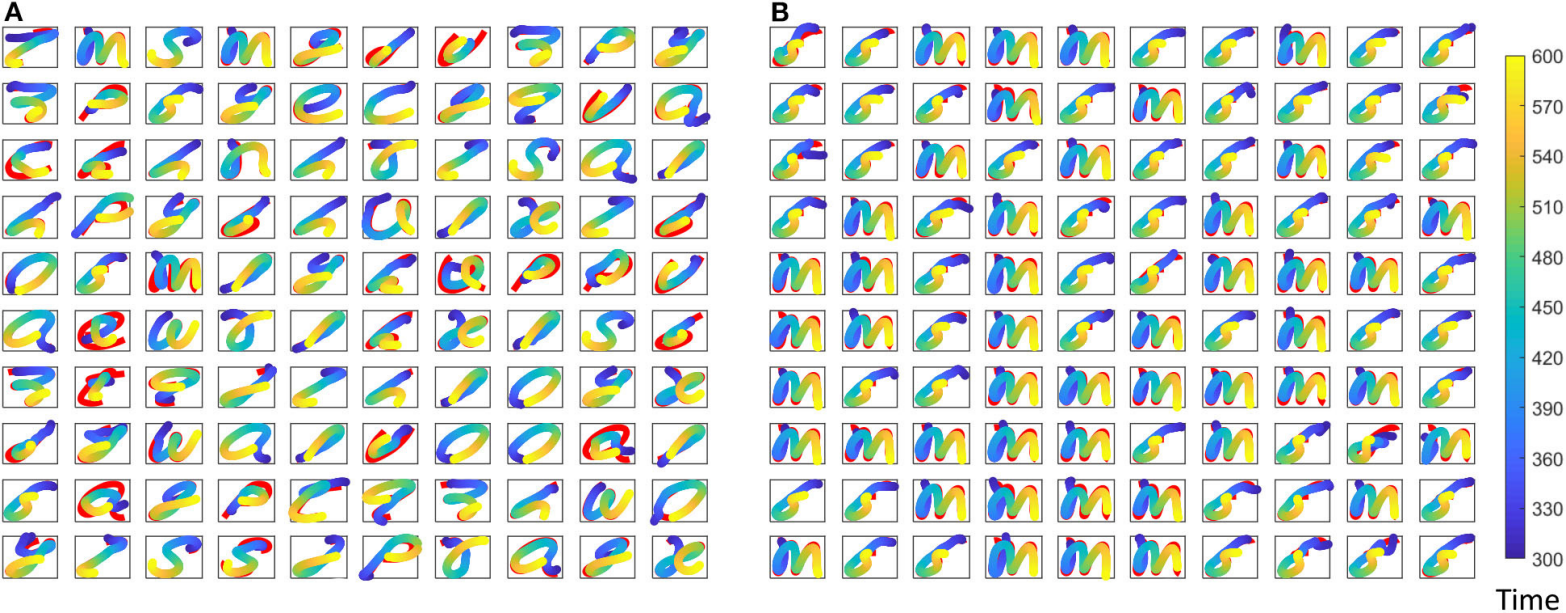
Different motor patterns generated by the ESN for randomly picked 100 input utterances from the TI-alpha test data set. **(A)** The spoken alphabet letter in the input instance. **(B)** The gender of the speaker with “f” for female and “m” for male. Color code shows the time evolution of the signal and shown in red is the expected motor pattern.

**Figure 9:**
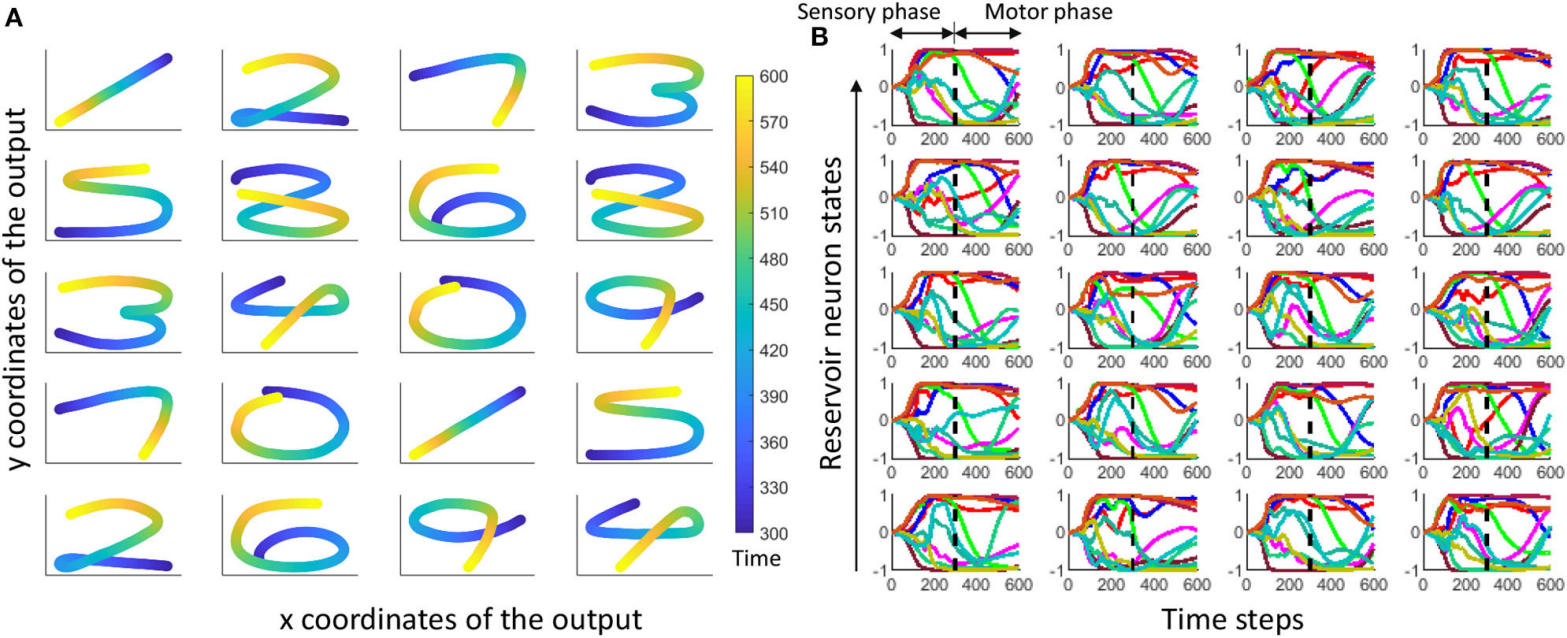
**(A)** The hand drawn ESN outputs for 20 randomly selected input voice signals from TI-10 testing dataset. Color elaborates the time evolution of the drawn impression. **(B)** Time evolution of reservoir neuron states for randomly picked 10 neurons (out of 400) corresponding to each output in **(A)**.

The target motor patterns are given in terms of temporally varying *x* and *y* coordinates. Hence there are two outputs (*x* and *y*) in the readout as shown in Figure 6. The figure shows how the *x* and *y* coordinates vary with time for a hand drawn impression of digit 6. All the spoken words (10 digits and 26 alphabet letters) were hand drawn in such a way that no lifting in the hand is required while drawing. The sequential coordinates of the images were extracted with the assistance of MATLAB *ginput*() function. The obtained *x* − *y* coordinates were then resampled to generate equally distant points. Same RLS rule is used for training the readout weights *w*_*out*_ :

(12) $$\begin{array}{r} w_{out}\left(t\right)=w_{out}\left(t-\Delta t\right)-P\left(t\right)r\left(t\right)e\left(t\right) \end{array}$$

where *r*(*t*) is the reservoir dynamics and *e*(*t*) gives the error between the expected coordinates and the actual coordinates at the output. Weight updates for the gender recognition is independently carried out from that of the spoken word recognition. Figure 7 shows expected and actual (red) impressions drawn for the TI-10 motor pattern generation task. Figure 8 shows expected and actual (red) impression drawn for the TI-alpha motor pattern generation task. The color of the motor pattern explains the time evolution of the coordinates at the readout. Figure 9 shows the corresponding reservoir dynamics of 10 randomly selected neurons during the sensory and motor phases for the TI-10 data set. Refer to the Supplementary Video to view the RNN drawing digits.

### 3.2. Separation vs. Accuracy

We measure the error by means of the average squared difference between the actual and the expected output (at the readout) per input instance in the testing data set. The error *d* is given by

(13) $$\begin{array}{r} d=\frac{1}{N_{ex}}\overset{N_{ex}}{\underset{i\;=\;1}{\sum }}\sqrt{\overset{N_{points}}{\underset{j\;=\;1}{\sum }}{\left(x_{i,j}^{t}-x_{i,j}^{a}\right)}^{2}+{\left(y_{i,j}^{t}-y_{i,j}^{a}\right)}^{2}} \end{array}$$

where *N*_*ex*_ is the number of examples in the test data set, *N*_*points*_ is the number of sample points in the output motor pattern, $x_{i,j}^{t}$ is the target *x* coordinate and $x_{i,j}^{a}$ is the actual *x* coordinate at the output of the jth point in the ith example. Similarly, $y_{i,j}^{t}$ and $y_{i,j}^{a}$ are target and actual *y* coordinates at the output, respectively. In the TI-10 digit drawing task, we noticed an average error of 0.0151, on the entire test data set. This is a ~37% reduction in average error with respect to a system without the class attractor separation step. We further evaluated the recognition accuracy of the ESN by means of an additional neural network.

The final output in our work is a motor pattern. To identify how well the digits were drawn, (i.e., can a human recognize the drawn character?), we used a Convolutional Neural Network (CNN). The CNN was pretrained to recognize hand drawn digits. The particular CNN used in the work has two convolutional layers followed by subsampling. Each convolutional kernel is of size 5 × 5 and there are 6 and 12 feature maps at the output of first and second convolutional layers respectively (28 × 28 − 6*c*5 − 2*s* − 12*c*5 − 2*s* − 10*o*; Palm, 2012). The training set for the CNN consists of the MNIST training data set ($T_{MNIST}^{Train}$), and the 10 target hand drawn digits involved in this work (*T*_*TARGET*_). Finally, the trained CNN was fed with the output motor patterns generated by the ESN (*T*_*MOTOR*_) to observe how “recognizable” they are by a CNN. For generating the *T*_*TARGET*_ and *T*_*MOTOR*_, we converted the *x*, *y* coordinates of the temporal sequences into a 28 × 28 image to match the configuration of the instances in $T_{MNIST}^{Train}$. The CNN network was capable of classifying the 10 training images (*T*_*TARGET*_) with 100% accuracy and MNIST testing data set with 98.9% accuracy. The accuracy on *T*_*MOTOR*_ was 98.6%. This accuracy is approximately similar to that reported in Goudar and Buonomano (2018) (also used a CNN for classification). However, it is noteworthy that there are few key differences in the setup involved in Goudar and Buonomano (2018). Table 2 summarizes these changes along with the performances.

**Table 2:** The comparison with reference work.

**Network type**	**Discrimination based training?**	**RNN type**	**Number of reservoir neurons**	**Number of novel testing examples**	**Number of speakers**	**Accuracy on 10 hand drawn digits (%)**	**Ability to classify speaker gender**
Goudar and Buonomano (2018)	No	ESN	4, 000	410	5	98.7	No
This work	Yes	ESN	2, 000	1, 594	16	98.6	Yes

As tabulated in Table 2, the work proposed in Goudar and Buonomano (2018) uses a network with 4, 000 neurons, and shows an accuracy of 98.7% on 410 examples across five speakers. Therefore, we have achieved similar accuracy to Goudar and Buonomano (2018) with a network of half the size as Goudar and Buonomano (2018), and on ~4 × larger number of examples (we used all the 1, 594 instances from the TI-10 testing data set), owing to the discrimination based training approach.

To further validate the effect of class attractor separation on accuracy, we used the TI-alpha data set which has more number of classes and examples. We observed an error of 0.0596 in the spoken word generation task and an error of 0.0413 in the letter generation task related to the gender of the speaker. Without the separation step, we noticed an error increment of 29% in spoken word generation task.

**Figure 10:**
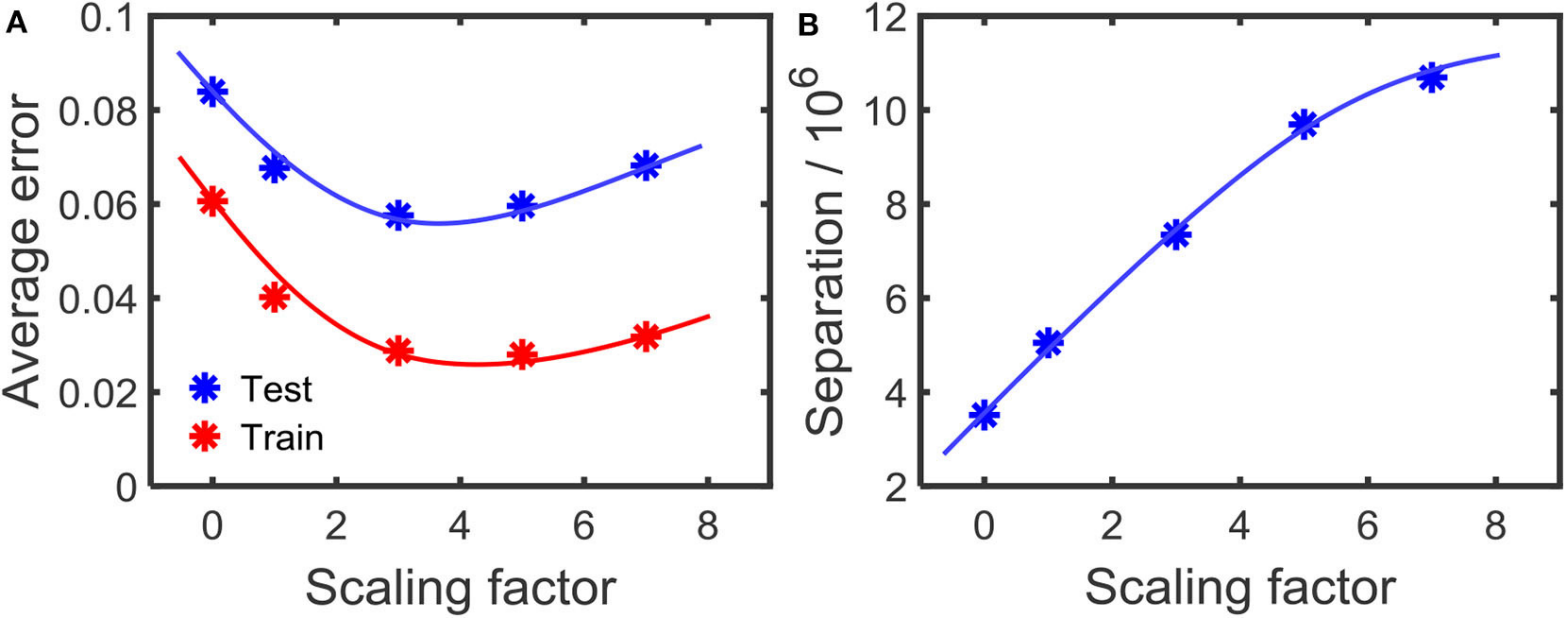
**(A)** The variation of the error with the amount of separation applied on the attractors. The separation increases with the scaling factor as shown in **(B)**. The results are for the TI-alpha, spoken word recognition application.

In order to observe the effect on the error at different amounts of separations, we changed the scaling factor γ in the first step of the learning process (Equation 9). Higher γ will increase the separation between the attractors. Figures 10A,B shows how the error changes with the amount of separation. As the figure illustrates, high separation leads to lower error. However, if the separation is too high, then the error increases. We conjecture that this is due to the inability to converge instances in a class to its corresponding attractor, due to the high separation. With high separation, the network enhances subtle changes in the input to an extent that the approximation step could no longer converge the inputs to their corresponding class attractor. This phenomenon is clearly explained in prior work (Wijesinghe et al., 2019), showing that the increased separation along with insufficient improvement in approximation property leads to reduced classification accuracy in reservoir computing.

Obtaining this optimum point (that gives the highest accuracy) before expensive training is beneficial. Multiple methods of identifying the optimum point based on separation and approximation for reservoir computing are available in literature (Wang et al., 2015; Wijesinghe et al., 2019). As proposed in Wang et al. (2015), the metric for optimum performance point can be obtained by the following equation

(14) $$\begin{array}{r} D=\frac{\sqrt{R_{S}-R_{G}}}{R_{S}} \end{array}$$

Where *R*_*S*_ is a metric for separation and *R*_*G*_ is a metric for generalization. *R*_*S*_ is the rank of the *N* × *m* matrix *M*_*S*_, which contains the continuous time states [*x*_*u*_1__(*t*_0_), ..., *x*_*u*_*m*__(*t*_0_)] of the reservoir as its columns (explained in section 3.1.1). Same aforementioned rank concept is used for measuring *R*_*G*_, but now on a different matrix *M*_*a*_. *M*_*a*_ consists of reservoir states *x*_*u*_*ij*__(*t*_0_) as its columns, which are measured by feeding jittered versions of *u*_*i*_ (*u*_*ij*_) to the reservoir. Unlike *R*_*S*_, lower rank of *M*_*a*_ (= *R*_*G*_) suggests better generalization. The *R*_*S*_ metric for our work is the rank of the matrix that contains the attractors sampled at *t* = *t*_0_, as its columns. Given that the attractors are equal to the number of classes, we need to find the rank of a 2000 × 20 matrix (for the voice based digit drawing application). As explained in section 3.1.1, the metric *R*_*S*_ is most likely equal to the number of classes (i.e., 20), since *N* ≫ *m*. Therefore, *R*_*S*_ is simply a constant and may not contain any useful information. Hence the metric *D* may not be applicable for our work. However, the Discriminant ratio (DR) proposed in Wijesinghe et al. (2019) is much general and applicable in finding the optimum point. The metric can be elaborated in the following equations.

(15) $$\begin{array}{r} DR=tr\left(S_{b}\right)tr{\left(S_{w}\right)}^{-1} \end{array}$$

(16) $$\begin{array}{r} S_{w}=\overset{L}{\underset{i\;=\;1}{\sum }}P\left(\omega_{i}\right)\hat{\Sigma_{i}} \end{array}$$

where *P*(ω_*i*_) is the probability of class ω_*i*_, $\hat{\Sigma_{i}}$ is the sample covariance matrix (Park and Park, 2018) for class ω_*i*_. *tr*(*S*_*b*_) is explained in Equations (5) and (6), as separation property (SP). As shown in Wijesinghe et al. (2019), the point at which this DR is a maximum, is the optimum accuracy point. As shown in Figure 10A, for the digit drawing problem, the highest accuracy point lies at scaling factor ≈3.7. Obtaining this point before the reservoir-output weight training can be done by using the aforementioned DR metric.

### 3.3. Convergence and Stability of the Network

In this section we are exploring the convergence of the training method and the stability of the trained system. The RLS learning method involved in this work was specifically proposed for recurrent neural networks with chaotic activity (such as the network used in this work). The other algorithms designed for RNNs are computationally demanding and do not converge under chaotic activity (Rumelhart et al., 1986; Abarbanel et al., 2008; Sussillo and Abbott, 2009). The factors that can potentially affect the convergence are the learning rate and the number of parameters available for optimization. With more parameters for optimization, we can attain more convergence (Wijesinghe et al., 2017). However, as explained in Bengio (2012), when the number of hyper parameters of a network is high, it becomes less general i.e., the network can predict the data in its training set with high accuracy, but it will likely fail to perform correctly for previously unseen inputs. Mechanisms have been proposed in literature to avoid such over-training situations including early stopping of training (Doan and Liong, 2004), adding stochastic noise (An, 1996) etc. These methods are still applicable to the training method proposed in this work as well.

**Figure 11:**
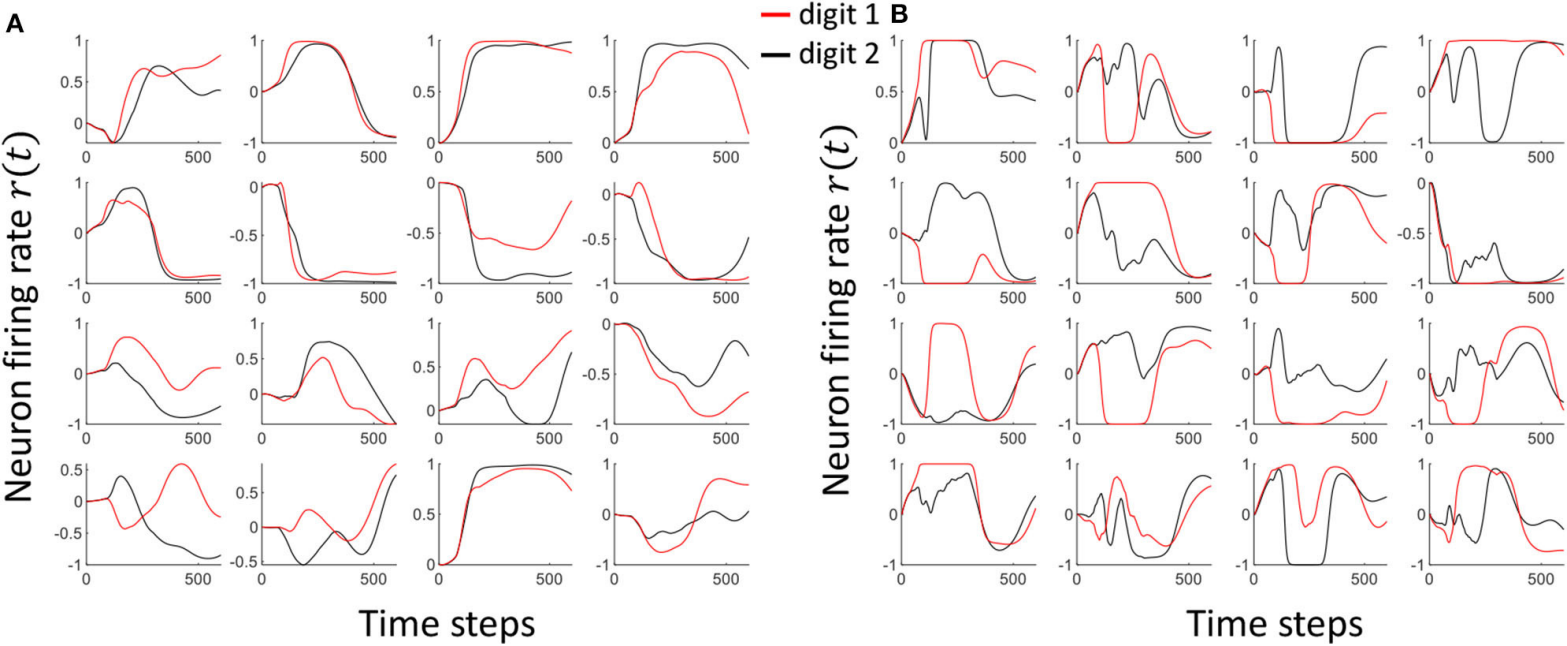
The Attractor dynamics corresponding to digit 1 and digit 2, in 16 randomly picked neurons, **(A)** before training **(B)** after training with a high scaling factor. Notice the increased amount of oscillations leading to much detailed attractors after training with high separation.

As shown in Equation (9), we are using a particular scaling factor during training. This scaling factor could potentially be viewed as the learning rate of the system. In general, high learning rates may hinder the convergence to a required solution. In fact, the output can oscillate between high accuracy and low accuracy states between epochs (Attoh-Okine, 1999). Smaller learning rates on the other hand would take more number of epochs to achieve convergence, and possibly reach a local minimum point rather than the global minimum. In this work, we have shown the effect on the scaling factor to the accuracy in Figure 10A. We have used the same number of epochs for the experiment. Therefore, the magnitude of the learning rate decides how much separation we apply between the attractors. High scaling factors lead to higher amounts of oscillations in attractors, while trying to reach higher separation. Figure 11 shows the attractors corresponding to digit 1 and digit 2 before (Figure 11A) and after (Figure 11B) training. The training was conducted with a high scaling factor (20). Note that the amount of oscillations has increased now to accommodate the separation between the attractors. Even though a good separation was achieved with the high separation rate, it is now difficult to converge different instances available in the same class to the same attractor (due to the highly detailed nature of the attractors).

**Figure 12:**
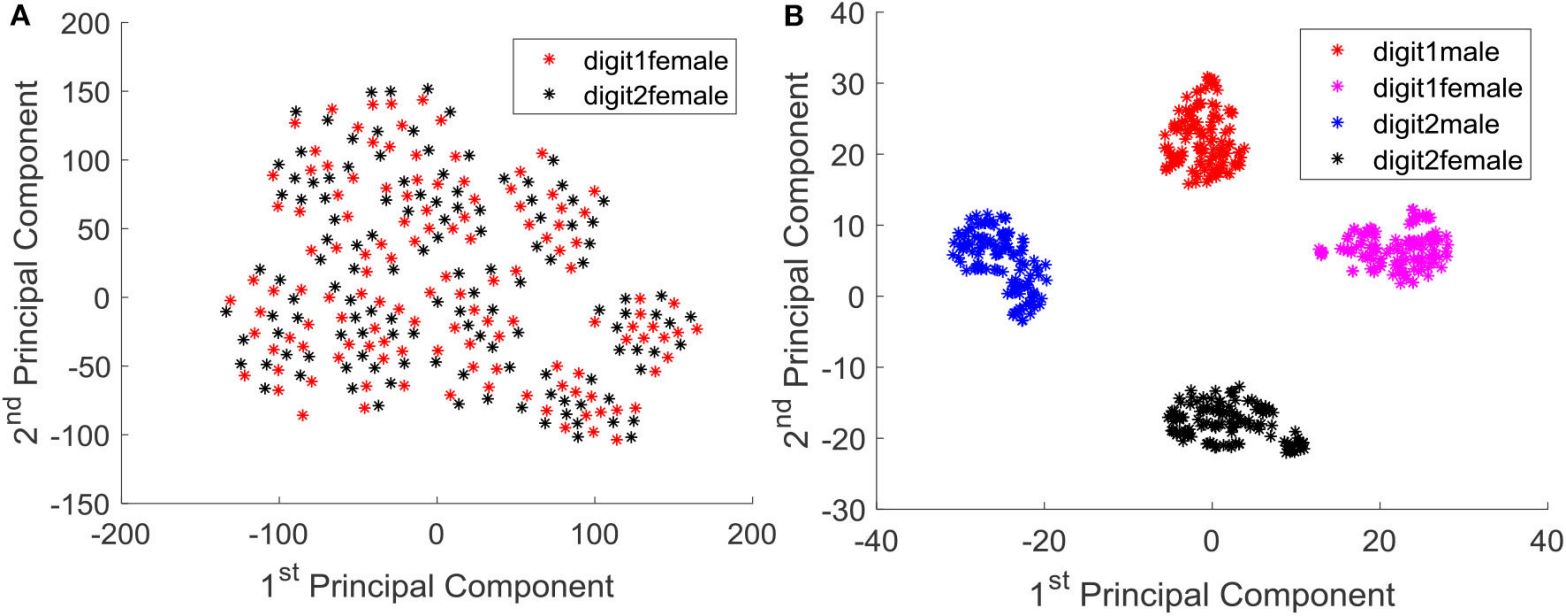
**(A)** Instances of class digit 1 and digit 2 (female) projected in to the 2D principal component space, before training. **(B)** Instances of class digit 1(male, female) and digit 2 (male, female) projected in to the 2D principal component space, after training.

We further analyzed the outcome of the trained system by means of principal components. Since the output is in high dimensional space, it is difficult to visualize the converged output. We concatenated temporal information of all the neurons in to a single vector per instance, and obtained the projection of them in to the two dimensional space with respect to the first two principal components. Figure 12A shows the data instances of two classes (digit 1 and digit 2) before training, and it is evident that the instances are not well separated and approximated. After training, the data instances are well separated and approximated as illustrated in Figure 12B. Note that even the male and female instances of each class are very well-separated.

**Figure 13:**
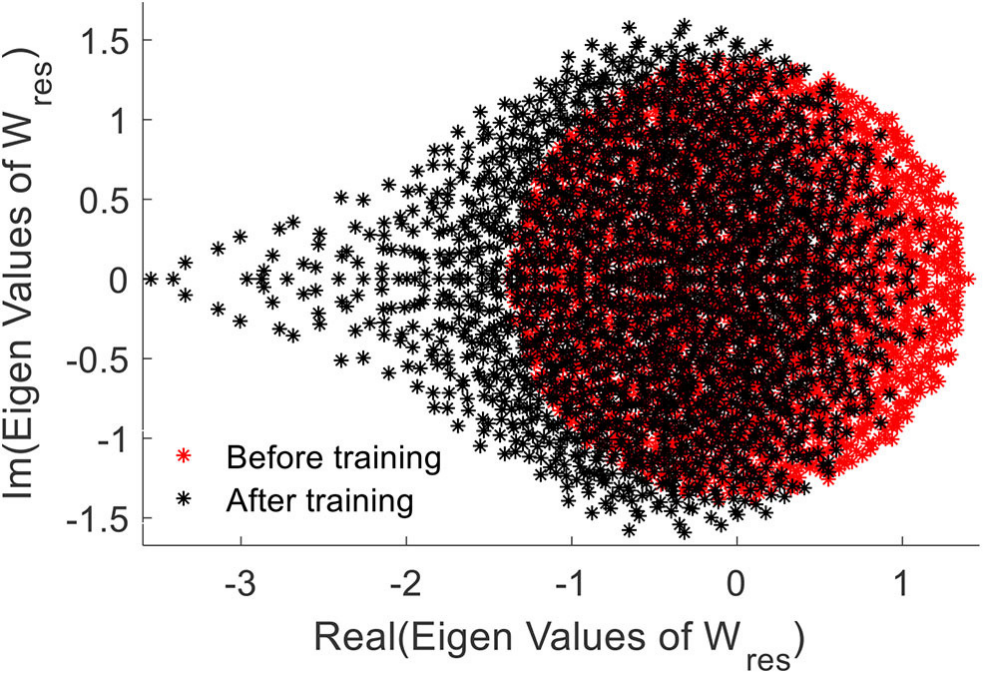
The Eigen value spectrum of the reservoir weight before (red) and after training the network.

We further elaborate the effect of training by means of the Eigen-value spectrum of the reservoir weight matrix. As explained in Rajan and Abbott (2006), the eigen values of the weight matrix of a network provides insights on stability of the network. We noticed that the eigen values of the trained weights are more compressed on the right hand side, when compared with the uniformly distributed initial eigenvalue spectrum (Figure 13). This elaborates increased stability of the network after the training (Rajan and Abbott, 2006).

## 4. Conclusion

Biological brain; a mystery yet to be solved, is not just a system that can be perceived as a simple cognitive machine. It has the capability to go beyond perception based inference, and is capable of interacting with multiple tasks. Cognitive and motor functions are interlinked in the brain (Leisman et al., 2016). Taking that as an inspiration, this work concatenates multiple tasks into a single network, creating a system that goes beyond perception. The learning algorithm furthermore tries to mimic the properties of the brain that allows massive amount of information to be stored. To enable efficient learning (memorizing), biological brain creates new connections among the existing memory structures. For an incoming input, the brain not only can observe how close it is to an existing memory anchor, but can also detect how different it is from another anchor. The learning rule explained in this work emulates such mechanisms of the brain to store information efficiently utilizing association (approximation) and dissociation (separation) between the data.

Our technique allowed to store twice the number of classes, with a reservoir of half the size (number of neurons), to achieve the same accuracy reported in Goudar and Buonomano (2018), on the entire TI-10 data set [which has ~4 times the amount of data than Goudar and Buonomano (2018)]. We further verified the accurate performance on an even bigger data set (TI-alpha) with 52 classes and 6, 628 training examples (in contrast to 20 classes and 2, 542 training examples).

Biological brain does not store everything in one learning process. Over time it learns new meaning, forgets unwanted information, and gets reshaped by experience. In contrast, our proposed algorithm assumes that all the data are available at the time of training. i.e., it does not learn one instance completely and move to the rest of the data. However, the algorithm can potentially be extended to learn things over time. It will be analogous to increasing the number of attractors over time, rather than starting with a predefined number of attractors. It is as if a baby learns mothers voice first (which is an attractor), and then over time the baby learns different speakers (more class attractors). The class attractors must be adjusted over time using separation, in order to make room for new data and create the dynamic dictionaries the biological brains have.

## Data Availability Statement

The datasets generated for this study are available on request to the corresponding author.

## Author Contributions

PW and CL performed the simulations. All the authors contributed in developing the concepts, generating experiments, and writing the manuscript.

## Conflict of Interest

The authors declare that the research was conducted in the absence of any commercial or financial relationships that could be construed as a potential conflict of interest.
