# Supplementary material for: Biologically Plausible Class Discrimination Based Recurrent Neural Network Training for Motor Pattern Generation
Source: Front Neurosci. 2020 Aug 12;14:772. doi: 10.3389/fnins.2020.00772 (PMC7461996; doi:10.3389/fnins.2020.00772)
Supplement: Supplementary file 2 [file Image_1.pdf]

Biologically Plausible Class Discrimination Based Recurrent Neural Network Training for Motor Pattern Generation - Supplementary material

Parami Wijesinghe [1,*,†] **Chamika Liyanagedera** [1,†] **and Kaushik Roy** [1]

[1] *Purdue University, School of Electrical and Computer Engineering, West Lafayette, Indiana, 47907 USA*

Correspondence*:
Parami Wijesinghe
pwijesin@purdue.edu

In order to show the generality of our training method, we are including here a third application that does not involve voice as an input command. In this application, the input is a hand drawn image, and the output is a time sequence that can be used to draw the corresponding digit. It further generates a letter 'i' or 'n' as another output at the same time, depending upon the face of the drawn digit ('i' for italic, 'n' for normal character face).

A subset (500 images) of MNIST data set was selected as the input images. Each image that has 28×28 pixels were converted into sequential temporal outputs from 28 input channels. Each channel produces a single pixel row of the image (up-sampled by $10\times$) as a time varying signal. Each input image is manually labeled by the face of the character: either italic or normal face.

At the end of the sensory phase, the residual dynamics in the reservoir are converted to time varying signals at the output by means of a readout layer. The readout layer gives two sets of time varying x and y coordinates of hand drawn impressions. One x and y output coordinate set produces a letter 'i' or 'n' indicating the input character face. The other output coordinate set produces the hand drawn impression of the character itself.

Figure 1 shows the expected and actual (red) impression drawn for the MNIST motor pattern generation task. The colors of the motor pattern explains the time evolution of the coordinates at the readout. For this MNIST image based application, we observed an average error of 0.0474 for generating the impression of the input digit, and an average error of 0.0296 for the motor pattern generation corresponding to the font face. Without increasing the separation between the attractors, we observed a 32.4% increment in error.

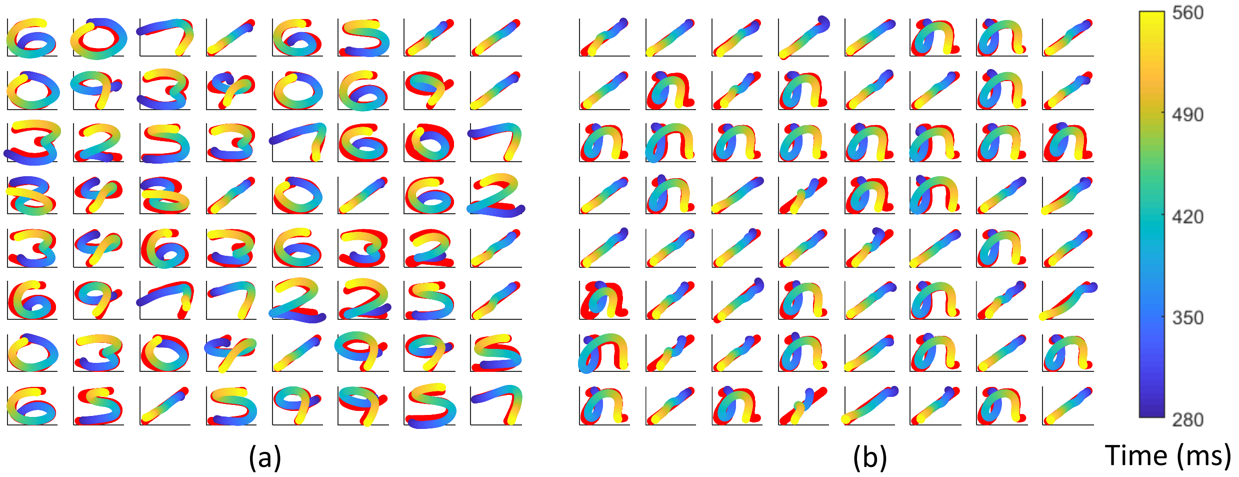

Figure 1. Different motor patterns generated by the ESN for randomly picked 64 input images from the MNIST test data set. (a) The hand drawn impression corresponding to the character included in the input instance. (b) The hand drawn impression corresponding to the character face. Letter 'i' represents that the input image was italic and letter 'n' shows that the input image was of normal font face. Color code shows the time evolution of the signal and shown in red is the expected motor pattern.
